# Diamond Like Carbon Films Containing Si: Structure and Nonlinear Optical Properties

**DOI:** 10.3390/ma13041003

**Published:** 2020-02-23

**Authors:** Šarūnas Meškinis, Andrius Vasiliauskas, Mindaugas Andrulevičius, Domantas Peckus, Sigitas Tamulevičius, Karolis Viskontas

**Affiliations:** 1 Institute of Materials Science, Kaunas University of Technology, K. Baršausko St. 59, LT-51423 Kaunas, Lithuania; andrius.vasiliauskas@ktu.lt (A.V.); mindaugas.andrulevicius@ktu.lt (M.A.); domantas.peckus@ktu.lt (D.P.); sigitas.tamulevicius@ktu.lt (S.T.); 2 JSC Ekspla, Savanorių 237, LT-02300 Vilnius, Lithuania; karolis.viskontas@gmail.com

**Keywords:** diamond-like carbon films containing Si, reactive magnetron sputtering, hexamethyldisilane, X-ray photoelectron spectroscopy, Raman spectroscopy, nonlinear optical properties, transient absorption spectroscopy

## Abstract

In the present research diamond-like carbon (DLC) films containing 4–29 at.% of silicon were deposited by reactive magnetron sputtering of carbon target. Study by X-ray photoelectron spectroscopy revealed the presence of Si–C bonds in the films. Nevertheless, a significant amount of Si–O–C and Si–O_x_ bonds was present too. The shape of the Raman scattering spectra of all studied diamond-like carbon containing silicon (DLC:Si) films was typical for diamond-like carbon. However, some peculiarities related to silicon doping were found. Studies on the dependence of DLC:Si of the optical transmittance spectra on the Si atomic concentration have shown that doping by silicon affects linear, as well as nonlinear, optical properties of the films. It is shown that the normalized reflectance of DLC:Si films decreased with the increased exciting light fluence. No clear relation between the normalized reflectance and photoexcited charge carrier relaxation time was found. It was suggested that that the normalized reflectance decrease with fluence can be related to nonlinear optical properties of the hydrogenated diamond-like carbon phase in DLC:Si film.

## 1. Introduction

Diamond-like carbon (DLC) is a metastable form of amorphous carbon and can be considered as some kind of nanocomposite, where sp^2^ bonded (graphite-like) carbon nanoclusters are embedded into the sp^3^ bonded (diamond-like) carbon matrix. It is estimated that 0–40 at.% of hydrogen may be present in these films [1,2,3]. Diamond-like carbon films are under considerable interest due to a very interesting combination of the high hardness, low friction coefficient, wear and corrosion resistance, and biocompatibility [1,2,3,4,5,6]. Optical and electrical properties, as well as surface-free energy of DLC films, can be varied in a broad range [1,2,3]. Properties of DLC films can be additionally controlled by introducing chemical elements other than carbon or hydrogen [1,3,4,5,6,7,8]. Particularly, silicon is one of the most often used DLC dopants [6,7,8,9,10,11,12,13,14,15,16,17,18,19,20,21,22,23]. Reduced internal stress and friction coefficient, as well as increased thermal resistance, while keeping high hardness of Si doped diamond-like carbon films were reported [9]. DLC films containing silicon are widely used for tribological and biomedical applications [6,7]. Silicon doping of DLC films resulted in reduced internal stress, increased hardness, and improved high-temperature tribological properties [15]. Reduced internal stress and friction coefficient of DLC:Si films as compared with undoped DLC films are presented in [16,19]. It was shown that friction coefficient of DLC:Si films in ambient air was lower than the friction coefficient of the undoped DLC films [14]. The incorporation of silicon was found as an efficient measure to improve the resistance of the DLC films against corrosion in HNO_3_ [13]. Due to high hardness, improved adhesion with Ti6Al7Nb substrate in comparison with undoped DLC and decrease of the human osteosarcoma cells proliferation, as well as adhesion of bacterial cells, DLC:Si films are suitable for creation of biologically and mechanically stable implants [12]. It was found that doping of DLC films by silicon may provide a protein adsorption control on the surface [21]. Si doping had effects on optical properties of DLC:Si films too. Increased transparency of hydrogenated DLC films as a result of silicon introduction was observed [10,11].

Along with widely studied mechanical, optical, and electrical properties of DLC films, other physical properties, such as nonlinear optical properties, are considered. The main two groups of the nonlinear optical behavior are self-saturable absorption (i.e., increase of a light transmittance with increased light beam intensity) and reverse saturable absorption (i.e., decrease of a light transmittance with increased light beam intensity) [24,25,26]. Recently, self-saturable absorption has been observed in diamond-like carbon nanocomposite films with embedded copper nanoparticles [24]. In addition, self-saturable absorption was found in some carbon-rich silicon carbide films [25]. Thus, a self-saturable absorption effect can be expected in DLC:Si films as well. Room temperature deposited hard, wear- and corrosion-resistant DLC-based saturable absorption mirrors could be serious competitors to the semiconducting absorber mirrors (SESAM), presently used for mode-locking of the ultrafast fiber lasers. It should be mentioned as well that semiconducting self-saturable absorption mirrors are grown at elevated temperatures by molecular beam epitaxy technique, a costly and complex thin film growth method. SESAM’s are a bulky, inflexible, and small operation wavelength range solution for optical fiber lasers [26].

Therefore, in the present study, DLC:Si films of different composition and structure were deposited by reactive magnetron sputtering of carbon target seeking possible relations between the structure, chemical composition, as well as linear and nonlinear optical properties.

## 2. Materials and Methods

DLC films were deposited by unbalanced reactive magnetron sputtering of graphite target. The diameter of the target was 3”. Si_2_(CH_3_)_6_ (hexamethyldisilane-HMDS) (Alfa Aesar, Tewksbury, MA, USA, catalog No A13155) vapor was used as a source of silicon and hydrogen. The vapor was introduced by using a liquid vaporizer (Bronkhorst High-Tech B.V., Ruurlo, The Netherlands). Argon was used both as sputtering gas and as transport gas. The base pressure in the vacuum chamber was 6 × 10^−4^ Pa. Deposition process work pressure was (7 ± 1) × 10^−1^ Pa. The gap between the substrate and the target was 0.1 m. In all cases, samples were grounded and no additional bias was used. The deposition time was ~2 min.

It should be mentioned that in the present study diamond-like carbon films were deposited by hexamethyldisilane vapor, a reagent containing silicon and no oxygen. Thus, deposited films are called DLC films containing silicon (DLC:Si).

Other deposition details of DLC:Si films are presented in Table 1. The conditions were varied to grow DLC:Si films containing different amounts of silicon.

The DLC:Si films deposited on Si (100) substrates were studied by X-ray photoelectron spectroscopy and Raman scattering spectroscopy. Samples for the study of the static and dynamic as well as linear and nonlinear optical properties were synthesized on fused silica substrates. The thickness of the samples was 50–90 nm.

The thickness and refractive index of the deposited films were measured by a laser ellipsometer Gaertner L115 (*λ* = 633 nm, Gaertner Scientific Corporation, Skokie, IL, USA).

Raman scattering spectra were measured by a Raman microscope inVia (Renishaw, Wotton-under-Edge, UK). Additionally, 532 nm wavelength excitation was used. Excitation power was set at 1.5 mW. The integration time was set at 100 s. The grating with a groove density of 2400 grooves/mm was applied. The main spectral feature of the Raman scattering spectra was fitted by two Gaussian form components, G peak and D peak. X-ray photoelectron spectroscopy (XPS) peak v3.1 software (Version 3.1, free software written by Ramund Kwok) was used for fitting. The possible presence of other peaks was not considered in this approximation.

A Thermo Scientific ESCALAB 250Xi spectrometer (Thermo Fisher Scientific, East Grinstead, UK) with a monochromatic Al Kα radiation (hν = 1486.6 eV) was used for X-ray photoelectron spectroscopy (XPS) measurements. The base pressure in the analytical chamber was lower than 2 × 10^−7^ Pa. The 40 and 20 eV pass energy values of a hemispherical electron energy analyzer were used for survey and high-resolution spectra respectively. The energy scale of the system was calibrated with respect to Au 4f_7/2_, Ag 3d_5/2_, and Cu 2p_3/2_ peak positions. For data analysis, the Thermo Scientific Advantage software (v5.979, Thermo Fisher Scientific, East Grinstead, UK) was used. All samples were analyzed before and after a gentle surface cleaning procedure using argon ions gun (500 eV, 50 sec).

Linear optical properties of the films were investigated using an optical spectrometer Avantes that is composed of a deuterium halogen light source (AvaLight DHc, Avantes, Apeldoorn, The Netherlands) and spectrometer (Avaspec-2048, Avantes, Apeldoorn, The Netherlands). The absorbance of the films was analyzed in the wavelength region from 200 to 1000 nm. Optical losses related to possible reflectance were not taken into account.

Ultrafast optical processes were studied by a transient absorption spectrometer HARPIA (Light Conversion, Vilnius, Lithuania). A more thorough description of the used measurement system can be found in [12].

The nonlinear optical response of the samples was researched by a nonlinear reflectance measurement system. Picosecond mode-locked fiber laser (JSC Ekspla, Vilnius, Lithuania) was applied as a variable wavelength light source (1020–1080 nm range). An excitation wavelength was 1064 nm. The nonlinear reflectance was also acquired at 1064 nm. For more information on the measurement setup used please see [24,27].

## 3. Results

### 3.1. Chemical Composition

**Figure 1:**
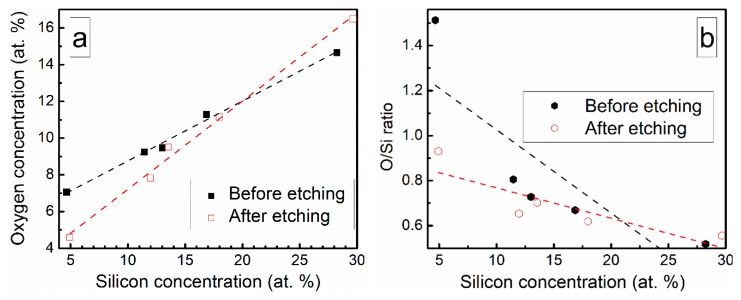
(**a**) Oxygen amount in DLC:Si films Vs Si atomic concentration and (**b**) O/Si atomic concentration ratio Vs Si atomic concentration.

Chemical composition of the deposited DLC:Si films is presented in Table 2. The DLC:Si films contain 4–29 at.% of silicon. However, in all films, oxygen is present and oxygen atomic concentration nearly linearly increases with Si amount in the films (Figure 1). Yet, O/Si atomic concentration ratio decreases with Si atomic concentration (Figure 1). In situ etching of DLC:Si films resulted only in minor changes in the chemical composition (Table 2, Figure 1). It should be mentioned, that an increase of the oxygen atomic concentration with Si amount in DLC:Si films was reported by other authors, too [12,18]. In numerous studies, relatively large amounts of oxygen, comparable with the present study or even higher, were reported for both hydrogenated [18,20,21] and hydrogen-free [12,17] DLC:Si films. In other case, O atomic concentration increased for DLC:Si films deposited by plasma-enhanced chemical vapor deposition from tetramethylsilane (TMS) only in comparison with the films deposited from the mixture of C_2_H_2_ and TMS [18]. This fact was explained by the reaction of TMS with oxygen [18]. Such a reaction can be additionally activated by plasma of the magnetron similarly to the silicon dioxide film deposition technology, where synthesis temperature can be significantly decreased by using plasma-enhanced chemical vapor deposition instead of the thermal chemical vapor deposition [28]. Thus, one can suppose that HMDS vapor reacts with residual oxygen in the vacuum chamber and we see increased oxygen content in DLC:Si films. In our case, deposition time was relatively short (~2 min.). Therefore, the source of residual oxygen may be water vapor desorbed from the vacuum chamber walls. In such a case, rapid reactions of the hexamethyldisilane with OH groups on the growing film surface and the resultant formation of –O–Si(CH_3_)_3_ can take place, as it was suggested by Soethoudt et al. [29].

**Figure 2:**
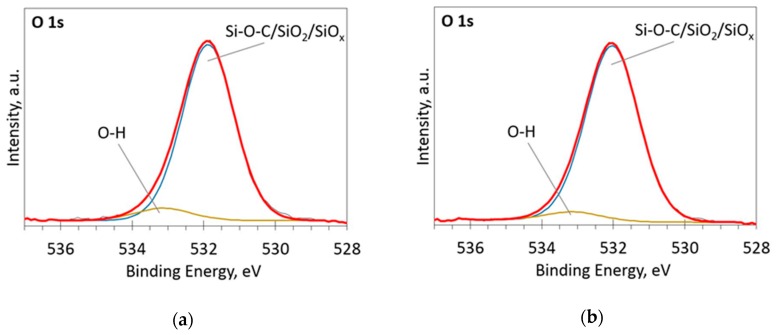
The deconvoluted high-resolution XPS spectra in O1s region (sample 954): (**a**) Before etching; (**b**) After etching: Thin black line—raw data, thick red line—envelope, other thick lines—fitted peaks.

**Figure 3:**
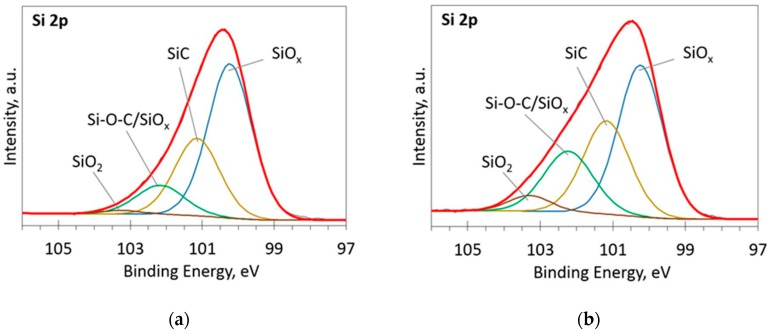
The deconvoluted high-resolution XPS spectra in Si 2p region (sample 954): (**a**) Before etching; (**b**) After etching: Thin black line—raw data, thick red line—envelope, other thick lines—fitted peaks.

**Figure 4:**
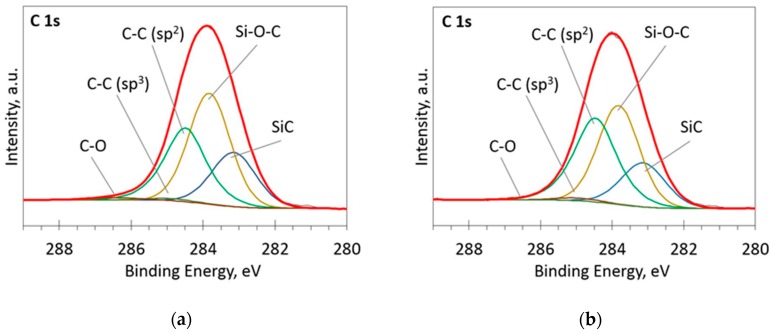
The deconvoluted high-resolution XPS spectra in C 1s region (sample 954): (**a**) Before etching; (**b**) After etching: Thin black line—raw data, thick red line—envelope, other thick lines—fitted peaks.

**Figure 5:**
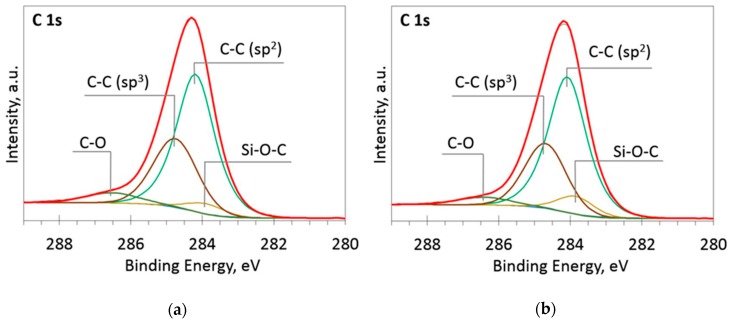
The deconvoluted high-resolution XPS spectra in C 1s region (sample 958): (**a**) Before etching; (**b**) After etching: Thin black line—raw data, thick red line—envelope, other thick lines—fitted peaks.

**Figure 6:**
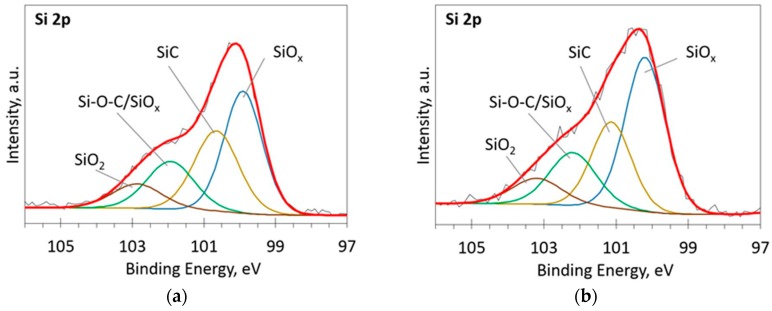
The deconvoluted high-resolution XPS spectra in Si 2p region (sample 958): (**a**) Before etching; (**b**) After etching: Thin black line—raw data, thick red line—envelope, other thick lines—fitted peaks.

The deconvoluted high-resolution XPS spectra in O1s, Si 2p and C 1s regions are presented in Figure 2, Figure 3, Figure 4, Figure 5 and Figure 6. In the case of the sample containing the largest amount of Si, the oxygen spectra consist of two peaks (Figure 2). They can be assigned to Si–OC/SiO_2_/SiO_x_ (~95%) and adsorbed O–H (~5%) bonds, since positions of these peaks (531.9 and 533.1 eV) correspond to the reported positions for SiO_2_ (532 eV) and O-H (533 eV) bonds [30]. The Si 2p region fitting resulted in four distinguishable peaks located at 100.2, 101.2, 102.2, and 103.3 eV on the binding energy scale (Figure 3). These positions are in good agreement with the known binding energy values for SiO_x_, SiC, Si–O–C, and SiO_2_ bonds [31].

**Figure 7:**
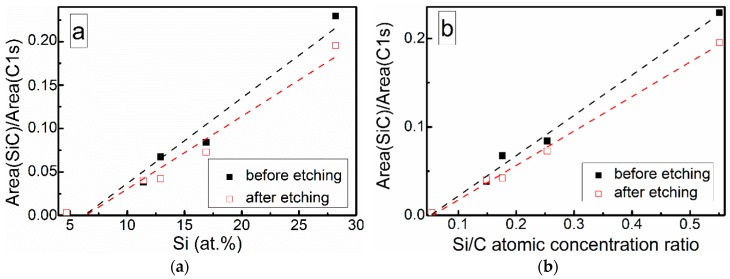
SiC fitting component and C1s peak area ratio vs. (**a**) Si atomic concentration and (**b**) Si/C atomic concentration ratio.

At low Si concentrations, SiC peak in the C1s region disappears (Figure 5), while in the Si 2p region, it stays almost the same (Figure 6). It can indicate that the SiC peak in Si 2p region is a mixture of at least two peaks: a) Peak corresponding to Si-C bonds, b) peak representing Si–Ox bonds. On the other hand, the SiC peak in the C1s region area ratio with C1s peak area quasilinearly increased with the Si atomic concentration (Figure 7a). As this ratio linearly increased with the Si/C atomic concentration for ion beam cleaned samples (Figure 7b) too, one can assume that SiC peak in the C1s region is related to the Si–C bonds only.

### 3.2. Structure

**Figure 8:**
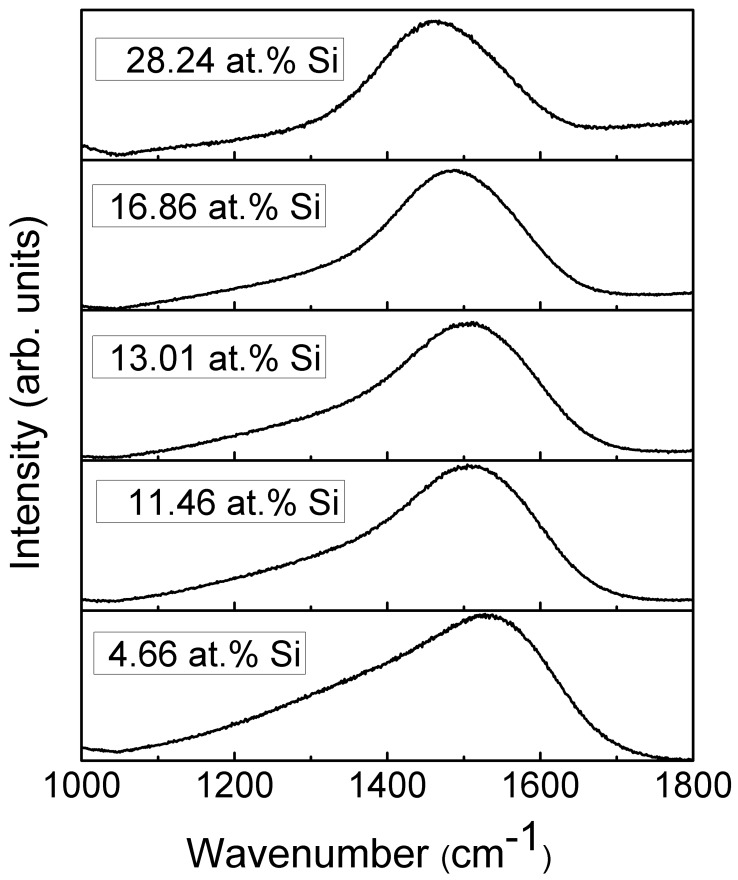
Representative Raman scattering spectra of DLC:Si films.

The representative Raman scattering spectra of the DLC:Si films are shown in Figure 8. The shape of spectra is typical for diamond-like carbon [1,32]. One can see a wide spectral feature in the 1200–1700 cm^−1^ wavenumber range. This feature is usually defined as a superposition of two peaks. In this case, the main peak at ~1400–1600 cm^−1^ is explained as a stretching vibration mode of sp^2^-bonded carbon atoms. It is called the G peak. While the shoulder in ~1200–1400 cm^−1^ wavenumbers range is explained as a disorder-induced D peak associated with the sp^2^-bonded carbon ring breathing mode [1,32].

**Figure 9:**
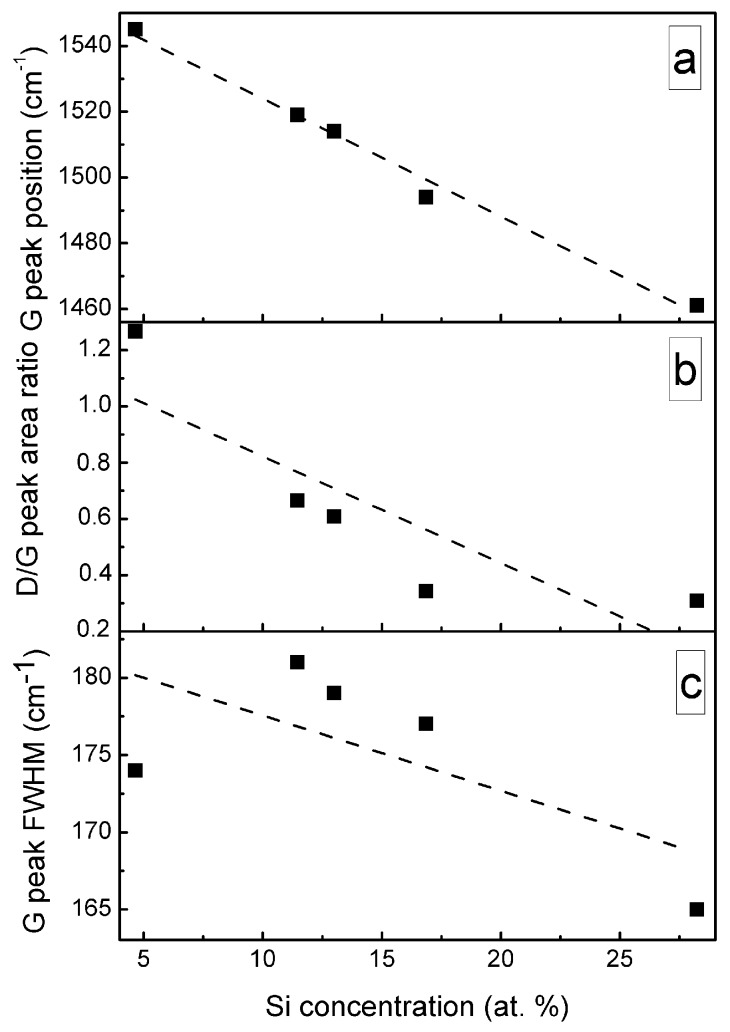
G peak position (**a**), D/G peak area ratio (**b**), G peak FWHM (**c**) Vs Si amount in DLC:Si film.

A significant, nearly-linear downshift of the G peak position with the increased Si atomic concentration should be mentioned (Figure 9). In the case of the samples containing a small amount of Si, the position of the G peak is close to that of the undoped DLC. While in the case of the sample containing 28.24 at.% of Si, position of the main peak at 1461 cm^−1^ is far below minimum G peak position values (1520 cm^−1^) found for the undoped diamond-like carbon, and even below minimum G peak position value reported in carbon amorphization diagram (1520 cm^−1^) [1]. At the same time, D/G peak intensity ratio decreased with Si atomic concentration in the film down to ~0.3. According to [1], such behavior of G peak position and D/G peak intensity ratio indicates a significantly increased sp^3^/sp^2^ carbon bond ratio. It should be mentioned that such behavior was reported in numerous studies for both hydrogen-free and hydrogenated DLC films containing silicon or SiO_x_ [8,33,34,35,36]. G peak position downshift to 1505 cm^−1^ [21], 1495 cm^−1^ [23], 1480 cm^−1^ [12] and even as low as 1460 cm^−1^ [17,19] was reported. In those studies, the changes were explained by the increased sp^3^/sp^2^ carbon bond ratio. However, it can be seen in Figure 9c that FWHM of G peak decreases with the increased Si atomic concentration. According to Ferrari et al. [32], sp^3^/sp^2^ carbon bond ratio decreases with the decrease of G peak FWHM. Thus, Figure 9a–c provides in some way contradicting information of changes in the amount of sp^3^ carbon phase. In addition, according to Komori et al. [17], the D/G ratio in the case of hydrogenated DLC:Si film is lower than the D/G ratio of the Raman spectra of tetrahedral amorphous carbon film. Thus, it should be supposed that sp^3^/sp^2^ carbon bond ratio in that case is higher in DLC:Si film than that in tetrahedral amorphous carbon film. It must be mentioned that sp^3^/sp^2^ carbon bond ratio in the case of the hydrogenated DLC films deposited by plasma-enhanced chemical vapor deposition was significantly lower than in the case of tetrahedral amorphous carbon films [1]. Taking into account the downshift of the G peak to the position below any value reported for undoped DLC film [1] mentioned above, the relation between the downshift of G peak position of DLC:Si films and increased sp^3^/sp^2^ carbon bond ratio seems to be doubtful. Bociaga et al. [12] and Batory et al. [23] explained downshift of G peak position by reduced internal stress. However, stress reduction as a result of DLC doping by chemical elements other than Si, C, H was reported in numerous studies. Yet that reduction was not accompanied by so significant downshift of G peak position. While, according to Zhu et al. [37], in the case of undoped DLC films, the downshift of G peak position was at the level of several cm^−1^, or no shift was found despite significant stress changes in 3 GPa range. Therefore, possible reduction of the internal stress in DLC:Si films cannot explain such a significant G peak downshift. It should be mentioned that at 1460–1470 cm^−1^ transpolyacetylene, Raman scattering peak is present [38]. In addition, in some cases in Raman scattering spectra of DLC:Si and DLC:SiO_x_ films other transpolyacetylene-related peaks were observed [8,35,36]. Besides, the study of DLC:SiO_x_ films by multiwavelength Raman scattering spectroscopy revealed the dependence of the spectra on excitation wavelength [39]. Transpolyacetylene-related spectral features can be seen in the case of the Raman scattering spectra excited by 633 and 785 nm wavelength light, while no transpolyacetylene related separate peaks or shoulders of the peaks were found in spectra excited by 532 nm wavelength light. Thus, it can be supposed, that in the case of the DLC:Si and DLC:SiO_x_ films, the main feature of Raman scattering spectra should be described by three peaks instead of two. The transpolyacetylene peak appears along with G and D peaks. In such a case, we see the increased intensity of the transpolyacetylene peak. Such a change can be wrongly interpreted as significant downshift of the G peak position if the main feature is fitted just by G and D peaks.

### 3.3. Optical Properties

**Figure 10:**
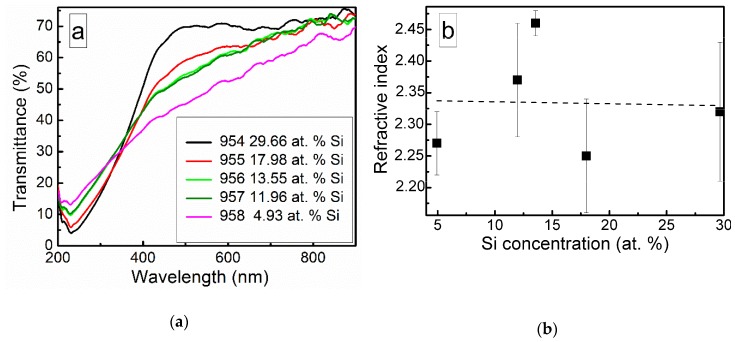
Optical transmittance spectra (**a**) and refractive index (**b**) of DLC:Si films.

Optical transmittance spectra of the DLC:Si films are shown in Figure 10. Doping of the DLC films by silicon resulted in a decreased optical transmittance in a 200–350 nm wavelength range. While in higher wavelengths range, increased optical transmittance was found.

It should be mentioned that other authors reported similar results. Much the same changes of the optical properties as a result of silicon doping were reported by Jedrzejczak et al. [11]. Si doping increased transparency of hydrogenated DLC films in numerous studies [10,40,41]. However, DLC:Si films contained up to 11 at.% O [11] and 13–22 at.% of oxygen [40]. Thus, in those cases, the possible influence of SiO_x_ should be taken into account, too. Yet, the bandgap of amorphous silicon carbide can be beyond 3 eV [42]. Thus, changes in optical properties can be explained by the presence of both SiC_x_ and SiO_x_ phases observed in the DLC:Si films (Figure 2, Figure 3, Figure 4, Figure 5 and Figure 6).

Refractive index of the deposited DLC:Si films measured at 632 nm wavelength was in 2.25–2.4 range. It should be mentioned that, in our previous study, refractive index of hydrogen-free undoped DLC films deposited by unbalanced magnetron sputtering was very similar [43]. Refractive index values 2.2–2.5 were found. Values of the refractive index of DLC:Si films similar to the present study were reported by Kato et al. [10] and Jedrzejczak et al. [11]. It was 2.1–2.2 [10] and 2.15–2.3 [11]. According to Kato et al. [10] and Jedrzejczak et al. [11], silicon doping can slightly increase the refractive index of DLC films. However, it can result in a significant decrease down to 1.8 of the refractive index if a significant amount of oxygen present in the film [31,40]. In addition, the presence of the hydrogen results in a reduction of the refractive index [1]. Thus, in our case, silicon doping has only minor effects on the refractive index of DLC films. No effects of the SiO_x_ phase observed by XPS (Figure 2, Figure 3, Figure 4, Figure 5 and Figure 6) and hydrogen can be found.

**Figure 11:**
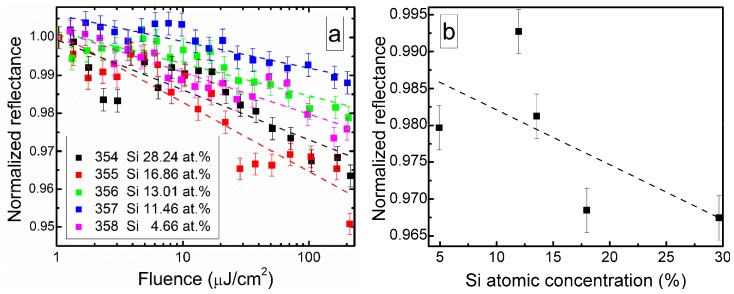
(**a**) Normalized reflectance of DLC:Si films vs. laser fluence and (**b**) reflectance measured at 100 µJ/cm^2^ fluence vs. Si atomic concentration.

Nonlinear optical properties of DLC:Si films are illustrated in Figure 11. In all cases, reflectance of the films decreased with the exciting light fluence (Figure 11a). Non-monotonous dependence of the reflectance on silicon atomic concentration in the film was found (Figure 11b). The smallest decrease of reflectance was observed for the DLC:Si film containing 12 at.% of Si, and the largest one for the film containing 29.7 at.% Si. It should be mentioned that C/Si ratio (1.82) for DLC:Si film containing 29.7 at.% of silicon was nearly the same (1.83) as reported by Cheng et al. [25] for carbon-enriched SiC film, which exhibited saturable absorption effect.

There are two possible explanations of the different nonlinear properties in these films with nearly identical C/Si atomic concentration ratio. On one hand, in the present study a larger amount of oxygen was found in the DLC:Si film, in comparison with study by Cheng et al. [25]. It was 16.49 and 2.1 at.%. On the other hand, no peaks typical for DLC in Raman scattering spectra of the film mentioned above were seen by Cheng et al. [25]. Thus, the differences between research of Cheng et al. [25] and present study can be explained by the presence of higher amounts of silicon oxycarbide and silicon oxide and/or by the different structure of the carbon phase. In our previous study [24], the reflectance of undoped hydrogenated DLC film decreased by ~2% at 230 µJ/cm^2^. Such value was similar to the reflectance decrease observed in the present study. Thus, it can be supposed that the normalized reflectance decrease with fluence is related to the nonlinear optical properties of the hydrogenated diamond-like carbon phase.

**Figure 12:**
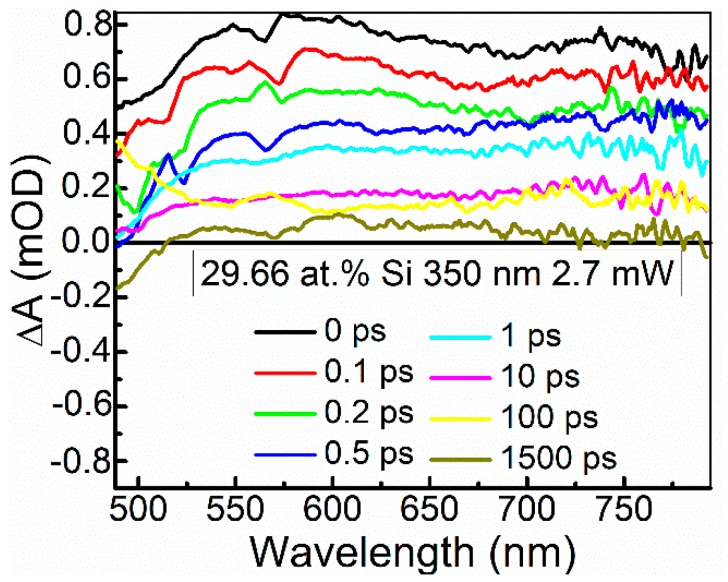
Typical transient absorption spectra of DLC:Si films.

**Figure 13:**
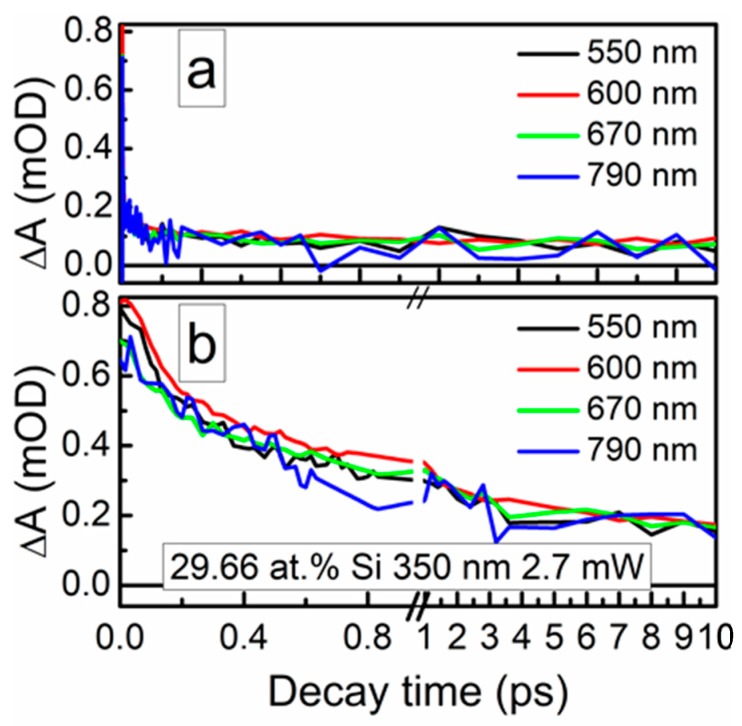
Typical TAS traces of the DLC:Si films.

The ultrafast optical processes were studied by transient absorption spectroscopy (TAS) to reveal possible relations between the nonlinear optical properties of the samples and their excited state relaxation times. It should be noted that in our previous study [24], saturable absorption was observed for the DLC:Cu films, when the photoexcited charge carrier relaxation time in these films was sufficiently high. Transient absorption signal amplitude (ΔA) of the DLC:Si films decreased with the increase of the excitation wavelength in 350–750 nm range. The strongest TAS signal was recorded for the spectra excited by 350 nm wavelength light (Figure 12 and Figure 13). In the case of higher excitation wavelengths, for most samples, TAS signal strength drop was too high for reliable analysis of the measurement data.

The photoexcited charge carrier relaxation times were calculated by fitting TAS signal relaxation decay traces. The exponential decay function with three components was used in Equation (1), like in [44,45,46]:

(1) $$I\left(t\right)=A_{1}e^{-\frac{t}{\tau_{1}}}+A_{2}e^{-\frac{t}{\tau_{2}}}+A_{3}e^{-\frac{t}{\tau_{3}}}+I_{0},$$

where *A*_1_; *A*_2_; *A*_3_ and *τ*_1_; *τ*_2_; *τ*_3_ are the amplitudes and time constants of decay, respectively, while *I*_0_ represents the TAS signal background.

**Figure 14:**
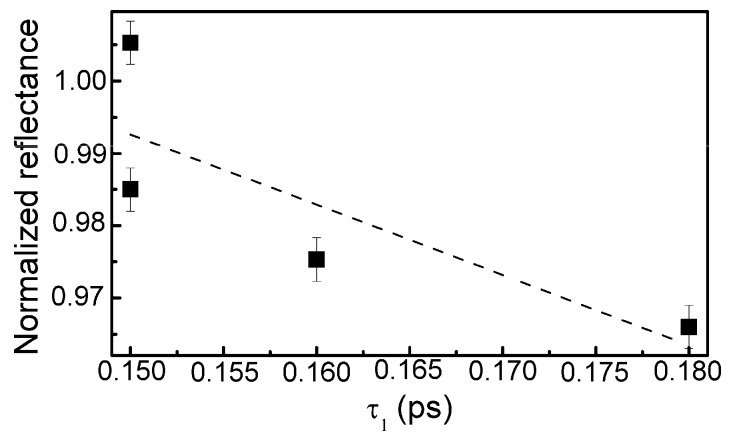
The normalized reflectance vs. relaxation time (relaxation time component).

The calculation data are presented in Table 3. In the case of the sample containing the lowest amount of silicon, reliable estimation of the relaxation times was impossible due to low amplitude of the TAS signal and high signal to noise ratio. Some correlation between the normalized reflectance and relaxation time (*τ*_1_) was found (Figure 14). However, in the present study relaxation times (*τ*_1_) were substantially lower than the relaxation times necessary for the appearance of the saturable absorption effect in the DLC:Cu films (0.15–0.18 ps vs. ≥ 0.5 ps).

## 4. Conclusions

In conclusion, DLC:Si films containing 4–29 at.% of silicon were deposited by reactive magnetron sputtering. Analysis of the XPS peaks revealed the presence of Si–C bonds in the film, however, a significant amount of Si–O–C and Si–O_x_ bonds was found in all studied films too. The shape of the Raman scattering spectra of all studied DLC:Si films was typical for diamond-like carbon and significant nearly-linear downshift of the G peak position with the increased Si atomic concentration was found. It was shown that the main feature of Raman scattering spectra should be described by three peaks (instead of usually used two), the transpolyacetylene peak appears along with G and D peaks. Doping of the DLC films by silicon resulted in decreased optical transmittance in 200–350 nm wavelength range, while in higher wavelengths range increased optical transmittance was found. Refractive index of the deposited DLC:Si films measured at 632 nm wavelength was in 2.25–2.4 range, such values are close to the refractive indexes reported for undoped DLC and DLC films containing silicon. The study of the nonlinear optical properties revealed a decrease of the normalized reflectance with the increase of exciting light fluence. Some correlation between the normalized reflectance and relaxation time (*τ*_1_) was found. It was supposed that the normalized reflectance decrease with fluence is related to the nonlinear optical properties of the hydrogenated diamond-like carbon phase.

## Figures and Tables

**Table 1:** Deposition conditions of DLC:Si films.

Sample No.	Ar Gas Flow (sccm)	Target Current (A)	HMDS Liquid Flow (mg/min)	Vaporizer Temp (°C)	Comments
954	140	0.62	2.5	40	In all experiments, HMDS liquid flow was the lowest available for the liquid vaporizer used.
955	140	0.9	2.5	40	
956	140	1.1	2.5	40	
957	140	0.9	2.5	20	
958	70 + 60	0.92	2.5	40	Ar gas flow was split into two equal fluxes. The first one was directed to the magnetron. The second one was used as a HMDS transport gas. Afterwards, Ar and HMDS mixture was introduced in the vacuum hamber through another inlet to increase the distance between the HMDS vapor inlet and sample.

**Table 2:** Chemical composition of the DLC:Si films (the presence of hydrogen in the films was not taken into account due to the method limitations.).

Sample No	Before Etching				After Etching			
	O (at.%)	C (at.%)	Si (at.%)	C/Si Ratio	O (at.%)	C (at.%)	Si (at.%)	C/Si Ratio
954	14.7	57.1	28.2	2.02	16.5	53.8	29.7	0.56
955	11.3	71.9	16.9	4.26	11.1	70.9	18.0	0.62
956	9.5	77.5	13.0	5.96	9.5	76.9	13.6	0.70
957	9.2	79.3	11.5	6.92	7.8	80.2	12.0	0.65
958	7.1	88.3	4.7	18.95	4.6	90.5	4.9	0.93

**Table 3:** Decay times of the samples calculated from TAS trace of wavelength at 600 nm. The excitation wavelength 350 nm.

Sample (Excitation)	*τ*_1_ (ps)	*A*_1_	*τ*_2_ (ps)	*A*_2_	*τ*_3_ (ps)	*A*_3_	R^2^
954 (350 nm)	0.18 ± 0.01	0.51 ± 0.01	1.97 ± 0.15	0.32 ± 0.01	65.7 ± 8.3	0.12 ± 0.01	0.997
955 (350 nm)	0.16 ± 0.01	0.46 ± 0.01	2.00 ± 0.18	0.29 ± 0.01	96.6 ± 13.3	0.13 ± 0.01	0.996
956 (350 nm)	0.15 ± 0.01	0.55 ± 0.02	3.19 ± 0.44	0.22 ± 0.01	137.8 ± 24.7	0.13 ± 0.01	1.000
957 (350 nm)	0.15 ± 0.01	0.48 ± 0.01	2.56 ± 0.38	0.21 ± 0.01	105.8 ± 16.8	0.15 ± 0.01	1.000
